# Global Dynamics of Grassland FVC and LST and Spatial Distribution of Their Correlation (2001–2022)

**DOI:** 10.3390/plants14030439

**Published:** 2025-02-02

**Authors:** Zhenggong Miao, Ji Chen, Chuanglu Wang, Shouhong Zhang, Yinjun Ma, Tianchun Dong, Yaojun Zhao, Rui Shi, Jingyi Zhao

**Affiliations:** 1 Beiluhe Observation and Research Station of Frozen Soil Engineering and Environment, Key Laboratory of Cryospheric Science and Frozen Soil Engineering, Northwest Institute of Eco-Environment and Resources, Chinese Academy of Sciences, Lanzhou 730000, China; miaozhenggong22@mails.ucas.ac.cn (Z.M.); zhaojingyi@lzb.ac.cn (J.Z.); 2 University of Chinese Academy of Sciences, Beijing 100049, China; 3 China Railway Qinghai-Tibet Group Co., Ltd., Xining 810007, China

**Keywords:** MODIS, grassland ecosystems, Fractional Vegetation Cover (FVC), Land Surface Temperature (LST)

## Abstract

Fractional Vegetation Cover (FVC) and Land Surface Temperature (LST) are critical indicators for assessing grassland ecosystems. Based on global remote sensing data for FVC and LST from 2001 to 2022, this study employs the Mann–Kendall trend test and Spearman correlation analysis to explore the dynamic changes in and spatial distribution patterns of both variables. The results indicate that the FVC is increasing in regions such as Europe, the eastern southern Sahara, western India, eastern South America, western and southern North America, and central China. However, it is decreasing in southern Canada, the central United States, and northern Australia. Significant increases in LST are observed in subarctic regions and the Tibetan Plateau, attributed to polar warming effects associated with global climate change. Conversely, the LST is decreasing in central China, eastern coastal Australia, and southern Africa. The global FVC–LST relationship exhibits the following four distinct spatial distribution patterns: (1) FVC increase and LST increase (Type 1), (2) FVC increase and LST decrease (Type 2), (3) FVC decrease and LST increase (Type 3), and (4) FVC decrease and LST decrease (Type 4). Type 1, covering 33.72%, is primarily found in high-latitude and high-altitude areas, such as subarctic regions and the Tibetan Plateau. Type 2, the largest group (46.98%), is mainly located in eastern North America, eastern South America, and southern Africa. Type 3, which comprises 18.72%, is concentrated in arid and semi-arid regions, while Type 4, representing only 0.59%, lacks clear spatial distribution patterns.

## 1. Introduction

The ramifications of global climate change for ecosystems, especially in highly sensitive grassland ecosystems, where its impact is notably pronounced, have attracted considerable attention [1,2,3]. Covering approximately 20% of the global terrestrial surface, grasslands play a pivotal role in regulating the global carbon cycle and mitigating climate change [4,5,6]. Two key indicators of ecosystem status, Fractional Vegetation Cover (FVC) and Land Surface Temperature (LST), can effectively reflect the responses of and changes in grassland ecosystems in the context of global warming [7,8]. FVC indicates the state of vegetation growth and the greenness of ecosystems [9,10], while changes in LST directly affect soil moisture, evapotranspiration, and plant physiological functions [11]. Investigating the dynamic relationship between FVC and LST not only helps to reveal the response mechanisms of grassland ecosystems to climate change, but also provides scientific evidence for climate adaptation and ecological conservation.

Variations in FVC exhibit substantial temporal and spatial disparities across diverse global ecosystems. In China’s Loess Plateau, there was a notable enhancement in the overall vegetation coverage from 2000 to 2016, with a marked surge between 2009 and 2016, witnessing a 14.16% rise compared to the period from 2000 to 2007. Nevertheless, this progression was not consistently distributed; the northern regions experienced a significant escalation in vegetation coverage, while parts of the northwest displayed a decline [12]. In the Qinghai–Tibet Plateau, spanning from 2017 to 2022, the overarching trend in vegetation coverage demonstrated a gradual ascent from west to east and from north to south. However, these changes were relatively subdued, with greater coverage in the southeast and diminished coverage in the northwest [13]. On a global scale, prominent alterations in grassland FVC were observed between 2001 and 2018, especially in specific areas of East Africa, South America, and Australia [14]. Humid and semi-humid regions typically present elevated FVC values, whereas arid and semi-arid regions display lower and more variable FVC values [15].

LST changes are a key indicator in the context of global climate change, and research in this field has become increasingly in-depth in recent years. Li et al. explored the lasting stability of LST changes influenced by climate change [16]. Some scholars believe that anthropogenic halogenated greenhouse gases are the main reason for the global warming hiatus from 2000 to 2015 and the recent rise in the global LST [17]. On a regional scale, Munawar et al. conducted research on LST changes in New Guinea Island, where the LST increased by 0.012 °C per decade from 2000 to 2019, but there were variations among subregions, with a significant decrease in the northwest and a significant increase in the south [18]. Another study focused on the Gomishan region of Iran, showing that the average LST reached about 42.5 °C in 2017, a significant increase from 33.8 °C in 1987 [19].

While regional and short-term studies have yielded insights into the relationship between FVC and LST, their limited scope constrains their applicability. For instance, early research in the grasslands of northeastern Kansas, USA, revealed a significant negative correlation between LST and the Normalized Difference Vegetation Index (NDVI), indicating that vegetation cover substantially influences LST [20]. In the Tibetan Plateau, satellite data analysis showed that forest expansion during dormant seasons reduced the LST [21]. Research on shrub encroachment demonstrated that changes in LST vary by regional conditions. Generally, LST increases in the semi-arid grasslands of the northern temperate zone but decreases in the humid regions of the southwestern United States [22]. On the global scale, studies analyzing the relationship between Green Vegetation Fraction and LST further revealed the impact of vegetation photosynthesis rates and water use efficiency on LST [23]. Long-term data analysis has been crucial in understanding the relationship between vegetation and temperature dynamics. For example, a long-term LST analysis from 2003 to 2017 based on the MODIS and ERA5 datasets showed a significant warming trend in northern latitudes. This was mainly caused by solar radiation and atmospheric longwave radiation [24]. In temperate grassland research, the asymmetric effects of daytime and nighttime temperature changes on vegetation coverage were revealed. Nighttime warming promotes FVC more effectively than daytime warming [25]. A subtropical spatiotemporal analysis of FVC from 2001 to 2018 confirmed significant growth trends that were closely related to precipitation and minimum temperatures, indicating regional differences in climate effects on vegetation dynamics [26].

In summary, while previous research has primarily centered on regional scales and short-term observations, yielding insights into the relationships between FVC and LST within specific ecosystems, their limited spatial and temporal scopes have constrained a comprehensive understanding of the long-term dynamics between FVC and LST. Addressing this limitation, our study adopts a global perspective on grassland ecosystems, leveraging a dataset that spans from 2001 to 2022. Through the application of Mann–Kendall trend analysis and Spearman correlation analysis, we aim to rigorously examine the trends and spatial distribution differences in FVC and LST changes. This investigation not only presents a novel exploration of the interrelationship between FVC and LST across global grasslands but also contributes vital insights for grassland management and the formulation of climate change adaptation strategies.

## 2. Results

### 2.1. Trend Variation

**Figure 1:**
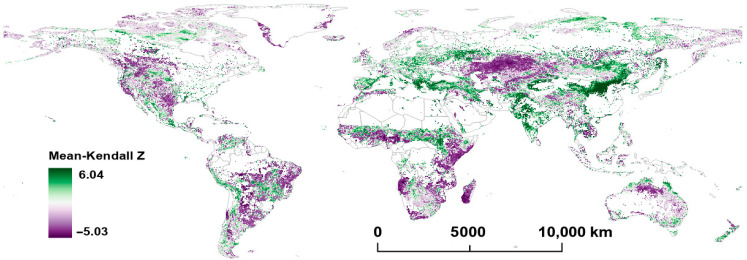
Per−pixel Mann−Kendall Z value of FVC from 2001 to 2022.

**Figure 2:**
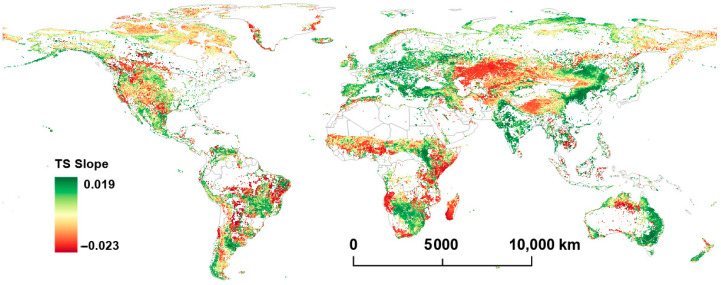
Per−pixel TS slope value of FVC from 2001 to 2022.

Figure 1 and Figure 2 illustrate the distribution characteristics of the Mann–Kendall Z (MK-Z) and TS slope of FVC in global grasslands from 2001 to 2022. From a global perspective, significant geographical distribution differences are observed in the MK-Z (*p* < 0.05) and TS slope (*p* < 0.05) of FVC.

FVC’s TS slope and MK-Z trends demonstrate similar patterns across various regions. In Europe, the eastern part of the southern Sahara Desert, eastern South America, western and southern North America, and central China, FVC exhibits an increasing trend. In contrast, in southern Canada, the central United States, and northern Australia, a decreasing trend of FVC appears in a scattered pattern, while its declining trend in West Africa and central Asia is relatively concentrated. In the Americas, the increase in FVC is not prominent, with a TS slope ranging from 0.0010 to 0.0030, while major increase areas are concentrated in central Brazil and Mexico, where the TS slope ranges from 0.0050 to 0.0110. In Europe, areas with notable FVC increases are mainly located on the northern shore of the Mediterranean, with a TS slope ranging from 0.0045 to 0.0078. In Africa, the southern part of the Sahara Desert shows a slight increase in FVC, with a TS slope ranging from 0.0015 to 0.0032, while in southern Africa and areas around South Sudan, FVC increases are more pronounced, with a TS slope ranging from 0.0035 to 0.0082. In Asia, a particularly significant FVC increase is observed in East Asia, especially central China, with a TS slope ranging from 0.0058 to 0.0118. Similar increases are seen in southeastern Australia, although on a smaller scale, with a TS slope ranging from 0.0035 to 0.0088. In contrast, northern Australia shows localized decreases in FVC, with a TS slope ranging from −0.0014 to −0.0032. In the Americas, areas of FVC decline are dispersed, with a TS slope ranging from −0.0017 to −0.0061. In Africa, FVC decline regions are both concentrated and scattered, encompassing central areas like Ghana and Nigeria (TS slope ranging from −0.0012 to −0.0028), eastern regions like Somalia, Kenya, and Tanzania (TS slope ranging from −0.0025 to −0.0048), and southern areas like Angola and Madagascar (TS slope ranging from −0.0028 to −0.0056). Central Asia, especially Kazakhstan and its surrounding areas, exhibits the largest concentration of FVC decline, with a TS slope ranging from −0.0014 to −0.0031.

**Figure 3:**
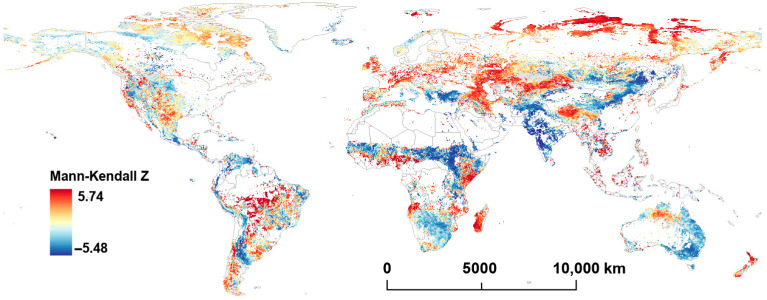
Per−pixel Mann–Kendall Z value of LST from 2001 to 2022.

**Figure 4:**
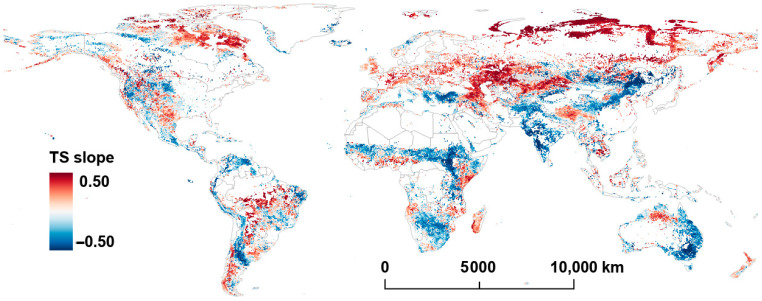
Per−pixel TS slope value of LST from 2001 to 2022.

Figure 3 and Figure 4 display the distributions of Mann–Kendall Z (MK-Z) and the Theil–Sen (TS) slope for LST from 2001 to 2022. Overall, there are significant geographical distribution differences in the MK-Z (*p* < 0.05) and TS slope (*p* < 0.05) of LST globally.

A large positive MK-Z and TS slope (TS slopes ranging from 0.18 to 0.26) are found across northern Asia, particularly near the Arctic Ocean, where LST has increased by at least 3.96 °C over the last two decades. Significant increasing trends in LST are also observed along the Eurasian–African continental boundary (TS slopes ranging 0.08 to 0.24). Furthermore, a large increasing trend is noticed over parts of central southern Africa and central South America, and small to moderate increases are observed in northeastern Canada and the western United States.

Notably, the Tibetan Plateau in China shows a significant rise in LST, with TS slopes ranging from 0.10 to 0.17. On the other hand, some regions, including the Andes Mountains in South America, the southern Sahara Desert in Africa, the Indian subcontinent, central China, and eastern Australia, display negative MK-Z values and TS slopes, indicating a relative decline in LST in these areas. Regions with more significant temperature declines (TS slope < −0.20) are represented in deep blue in Figure 4.

Comparing the trends in FVC and LST shows that there are often region-specific associations between the variables. For example, in central South America, central southern Africa, and Southeast Asia, both FVC’s MK-Z and TS slope are positive, indicating an increase in vegetation cover. In these regions, LST tends to show negative or relatively stable trends. This suggests a decoupling of vegetation and temperature increases, where vegetation expansion does not correspond to a rise in temperature.

In contrast, in northern Asia, the Tibetan Plateau, and the southernmost part of the Andes Mountains, both FVC’s MK-Z and TS slope are positive, and LST’s MK-Z and TS slope are also positive. This indicates that, in these areas, the increase in vegetation cover aligns with the rise in LST, exhibiting a consistent spatial distribution of both trends.

Considering these observations, it is imperative to investigate the correlation between FVC and LST. This will enhance our understanding of the interplay between vegetation changes and temperature variations across distinct regions.

### 2.2. Correlation Analysis

**Figure 5:**
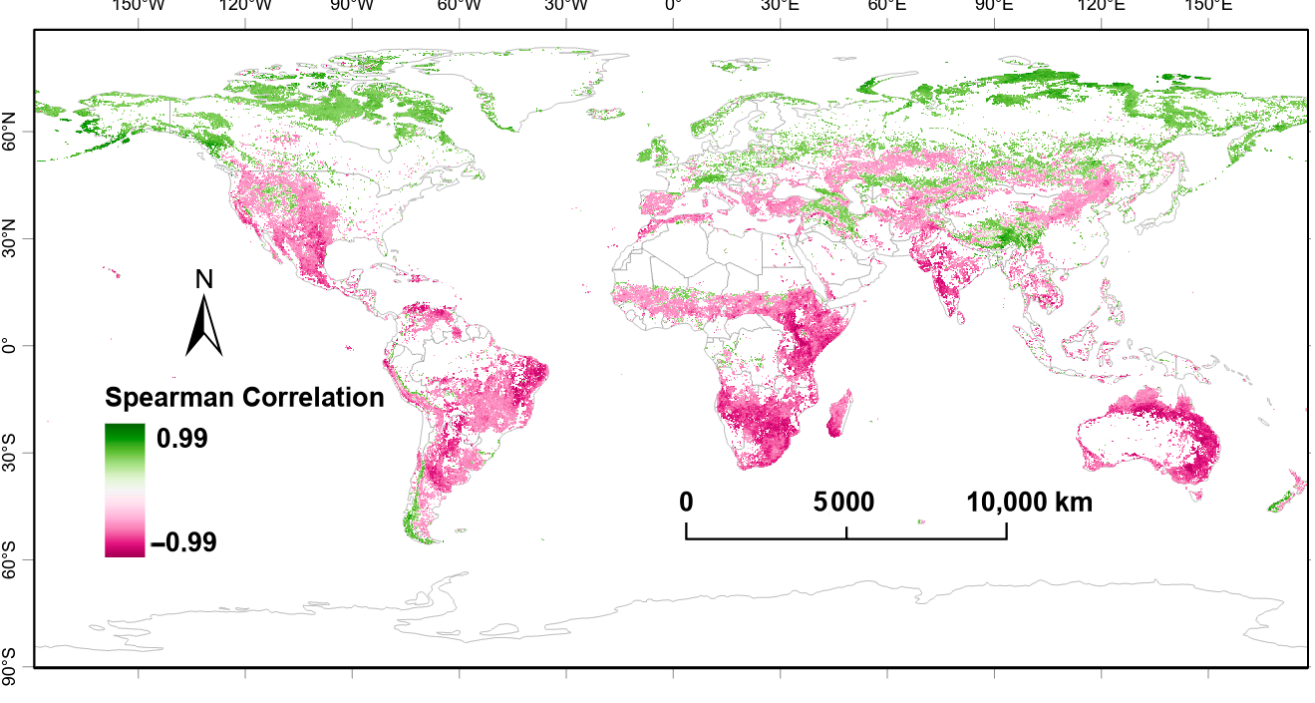
Spatial distribution of Spearman correlation coefficients for FVC and LST.

Figure 5 shows the spatial distribution of the Spearman correlation coefficients (*p* < 0.05) between FVC and LST, addressing the issue of the correlation between their trend changes. Different colors in the figure denote both the magnitude and direction of the correlation coefficient. Green indicates a positive correlation, where the trends of FVC and LST increase or decrease together, which typically occurs in high-latitude and high-altitude regions. Pink represents a negative correlation, suggesting that the trends of FVC and LST are opposite, and this is mainly distributed in tropical and subtropical regions, particularly in the central and eastern parts of South America, eastern and southern Africa, Australia, and the Indian subcontinent.

**Figure 6:**
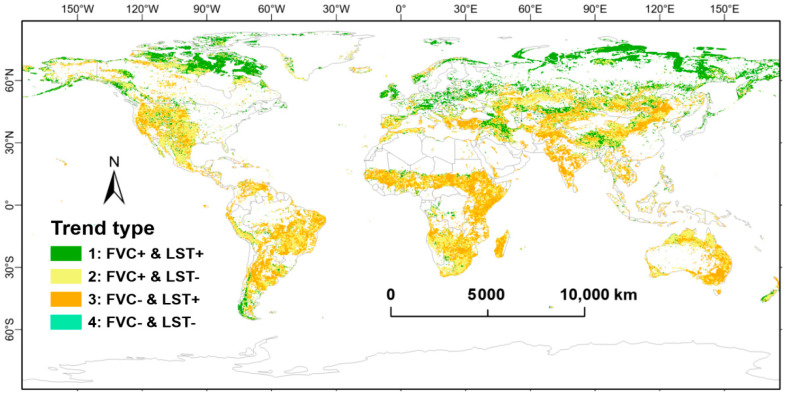
Spatial distributions of regions with significant positive and negative correlations for FVC and LST.

In order to more effectively delineate the spatial distributions of the varying increasing and decreasing trends in FVC and LST, the combinations of FVC and LST growth directions were categorized into the following four distinct classifications (Figure 6): FVC increase and LST increase (Type 1); FVC increase and LST decrease (Type 2); FVC decrease and LST increase (Type 3); and FVC decrease and LST decrease (Type 4).

**Figure 7:**
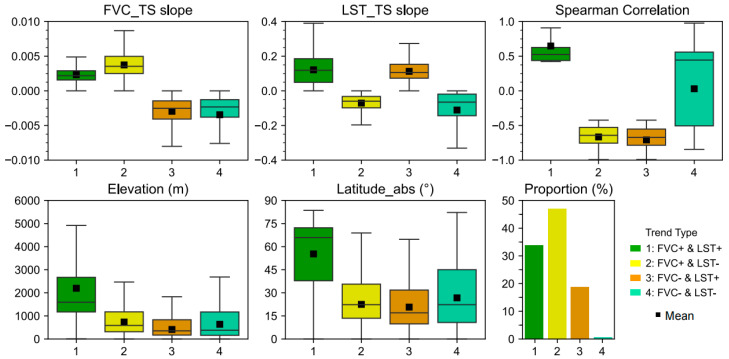
Distribution of data under different increasing and decreasing trends of FVC and LST.

Figure 7 illustrates the data distribution patterns for FVC_TS slope, LST_TS slope, Spearman correlation, Elevation, and Latitude_abs. Additionally, it depicts the proportion of each type. As shown in the proportion subplot of Figure 7, Type 2 has the highest proportion, accounting for 46.98%, while Type 4 has the lowest proportion, at only 0.59%. Types 1 and 3 account for 33.72% and 18.72%, respectively.

For the specific distribution of parameters, please refer to Table 1.

## 3. Data and Methods

### 3.1. Data Sources and Preprocessing

#### 3.1.1. Data Sources

The data for this study were primarily obtained through the Google Earth Engine (GEE) platform. GEE provides convenient cloud computing resources and a rich repository of remote sensing datasets, enabling large-scale spatiotemporal analyses. The specific datasets selected for this study are listed in Table 2.

The datasets used in this study include the MODIS NDVI (https://lpdaac.usgs.gov/products/mod13q1v061/, accessed on 26 July 2024), LST (https://lpdaac.usgs.gov/products/mod11a1v061/, accessed on 11 August 2024), Landcover (https://lpdaac.usgs.gov/products/mcd12q1v061/, accessed on 31 July 2024), and the Digital Elevation Model (DEM, https://dataspace.copernicus.eu/explore-data/data-collections/copernicus-contributing-missions/collections-description/COP-DEM, accessed on 16 August 2024). These datasets span the period from 2001 to 2022 and have varying spatial resolutions ranging from 30 m to 1000 m.

Preprocessing steps were applied to the downloaded data to ensure their quality and usability. These steps included resampling all data to a 1000 m resolution (each pixel covered an area of 1 km^2^ of the ground, or 1000 m × 1000 m), coordinate registration, cloud cover removal, and data reprojection. The representativeness and limitations of the datasets were considered in the analysis, and detailed descriptions of the data preprocessing steps, quality, and accuracy assessments are provided in the manuscript. Furthermore, all ethical and legal standards were adhered to throughout the study.

#### 3.1.2. Preprocessing

In the data preprocessing process of this study, the identification of grassland extent was based on the MODIS Landcover. Specifically, we extracted the spatial distributions of grassland cover types for each year from 2001 to 2022, and subsequently overlaid the grassland distribution layers for all years. Through overlay and frequency analysis, areas where grassland appeared more than 5 times or continuously existed for more than 3 years at the same coordinate points were designated as the grassland mask. This approach helped to exclude interference from short-term natural phenomena or human activities that could affect grassland identification, thus enhancing the robustness of the results and providing reliable spatial base data for subsequent grassland-related analysis. Based on this mask, the spatial ranges of NDVI, LST, and DEM for each year were further extracted to reduce the subsequent computational workload.

FVC was calculated using NDVI. NDVI, a commonly used indicator of vegetation growth status, ranges from −1 to +1 and can effectively reflect the health and growth condition of vegetation. To convert NDVI values into FVC values, an empirical formula-based method (Equation (1)) [19] was applied, which mapped NDVI values to the FVC range through a linear relationship. As a variable that more directly reflects vegetation cover, FVC provides more accurate and meaningful results.

(1) $$FVC=\frac{NDVI-{NDVI}_{min}}{{NDVI}_{max}-{NDVI}_{min}}$$

where NDVI_min_ values corresponding to bare soil are close to 0, while NDVI_max_ values for fully vegetated areas approach 1. However, remote sensing images are often affected by noise. Therefore, the minimum and maximum values of NDVI correspond to confidence intervals that should be selected based on specific conditions. In this study, the 5th and 95th percentiles of the lower and higher NDVI values, respectively, were chosen [27].

After completing the data clipping, a normality test was performed on the FVC and LST data for the period from 2001 to 2022 to ensure statistical validity for the regions used in subsequent analyses. Given the large dataset, the D’Agostino’s K-squared test [28] (also known as the D’Agostino–Pearson test) was selected. This method is widely used to assess whether data conform to the normal distribution assumption. D’Agostino’s K-squared test combines information about the skewness and kurtosis of the data to evaluate the degree of deviation of the sample data. Skewness measures the symmetry of the data distribution, while kurtosis measures the sharpness or flatness of the distribution. By examining these two statistical measures together, this test provides a comprehensive assessment of whether the data follow a normal distribution.

Since the data consisted of large-scale raster information, to improve computational efficiency and ensure test representativeness, 1,000,000 random pixel points were selected from each raster file for the normality test. This approach ensured that data processing efficiency was maintained while providing a sufficiently large statistical sample size to guarantee the reliability of the normality test results. The test results showed that both the FVC and LST data did not follow a normal distribution (*p* < 0.01), providing the basis for the selection of subsequent methods.

### 3.2. Trend Test and Estimator

The Mann–Kendall (MK) trend test and the Theil–Sen (TS) estimator are commonly used non-parametric methods for time series data analysis. They serve different purposes, but are often used together in trend analysis [29,30].

The MK trend test is designed to identify significant upward or downward trends in a time series without making assumptions about the specific form of data distribution. This test detects trends by assessing the signs of the differences between every pair of values in the sequence, and calculates the significance of the trend, typically presented in the form of a standardized statistic (Z-value). Specifically, a positive Z-value indicates an upward trend, while a negative Z-value indicates a downward trend. By standardizing the trend statistic into a Z-value, the significance of the trend can be evaluated. If the Z-value exceeds the critical value (*p* < 0.05), the trend is considered to be statistically significant [31,32]. The calculation of the Z-value is shown as follows:

The MK test determines the existence of trends at different time scales by calculating the MK statistic (S) (Equations (2) and (3)), as follows:

(2) $$S=\sum_{j=1}^{n-1}\sum_{i=j+1}^{n}sgn\left(x_{i}-x_{j}\right)$$

(3) $$sgn\left(x_{i}-x_{j}\right)=\left\{\begin{array}{c} -1,\;\;\;\;x_{i}-x_{j}<0\\ 0,\;\;\;\;\;\;\;x_{i}-x_{j}=0\\ 1,\;\;\;\;\;\;\;x_{i}-x_{j}>0 \end{array}\right.$$

where x_i_ and x_j_ represent data points at time i and j (i > j) and n is the total number of data points.

To quantify the significance of the trend from a statistical perspective, it is necessary to compute the probability related to S and the sample size nnn. For n ≥ 10, the statistic S is approximately normally distributed, with its mean and variance given by the following Equation (4):

(4) $$Var\left(S\right)=\frac{n\left(n-1\right)\left(2n+5\right)-\sum_{p=1}^{g}t_{p}\left(t_{p}-1\right)\left({2t}_{p}+5\right)}{18}$$

where n represents the number of data points, g is the number of sample datasets with the same value, and tp is the number of data points in the p-th group. For more details, refer to reference [33].

The standardized Z test statistic is calculated using Equation (5), as follows:

(5) $$Z=\left\{\begin{array}{c} \frac{S-1}{\sqrt{Var\;(S)}},\;\;\;S>0\\ 0,\;\;\;\;\;\;\;\;\;\;\;\;\;\;\;\;\;\;S=0\\ \frac{S+1}{\sqrt{Var\;(S)}},\;\;\;S<0 \end{array}\right.$$

While the MK test elucidates the direction and significance of trends within data, it does not directly quantify the intensity or rate of change of these trends. To address this limitation, the Theil–Sen (TS) estimator was developed. This estimator calculates the median of all possible slope values for pairs of data points (Equation (6)), thereby estimating the overall rate of change of the trend. Notably, the TS estimator is characterized by its robustness, particularly when dealing with outliers in data. By effectively mitigating the influence of outliers, it offers a more precise estimation of the trend slope compared to traditional linear regression methods [34,35].

(6) $$TS\;slope=Median\;\left(\frac{x_{i}-x_{j}}{t_{i}-t_{j}}\right)$$

where x_i_ and x_j_ represent the data values at times t_i_ and t_j_ (i > j).

The combination of the MK test and the TS estimator enhances the reliability of trend analysis, allowing for the revelation of the trend directions in time series data and the quantification of their rates of change. This combination enabled us to identify significant trends and estimate their specific rates of change, thereby providing a more comprehensive trend analysis. The MK test provides insight into the significance of the trend, while the TS estimator furnishes a quantitative slope estimate for the trend’s progression. This combination is particularly apt for time series data that deviate from a normal distribution and include outliers.

### 3.3. Correlation Test

In this study, to systematically assess the relationship between FVC and LST, we employed the Spearman correlation test. Spearman correlation is a rank-based non-parametric statistical method that calculates the correlations between variables by ranking the observed values, and it does not rely on the assumption of normality of data [36]. Unlike Pearson correlation, Spearman correlation is more robust when dealing with non-normal distributions, nonlinear relationships, or data with outliers, making it advantageous for handling the complexity of ecological data [37].

The Spearman correlation test allows for identifying the monotonic relationship between FVC and LST, which does not necessarily have to be linear, but rather assesses the trend between the ranks of the two variables. The correlation coefficient ranges from −1 to +1, where +1 indicates a perfect positive correlation, −1 indicates a perfect negative correlation, and values close to 0 suggest no significant correlation between the two variables. Specifically, we used Spearman correlation to explore the association between FVC and LST under different trend conditions, thereby revealing the potential ecological linkages between vegetation cover and LST change patterns.

## 4. Discussion

This study analyzes the MK-Z, TS slope, and Spearman correlation of the FVC and LST in global grasslands from 2001 to 2022, revealing the trends and significance of global FVC and LST changes. The findings reveal that FVC and LST demonstrate substantial differences in geographical distribution across the world. These disparities may be attributed to a multitude of factors, such as climate change, human activities, and dynamic changes in natural ecosystems.

### 4.1. Analysis of Regional Change Trends

From the content of Figure 1, Figure 2, Figure 3 and Figure 4, it can be seenn that in Europe, the eastern region of the southern Sahara Desert, western India, eastern South America, western and southern North America, and central China, there is an observed increasing trend in FVC. Along the comparatively arid southern edge of the Sahara Desert, the effects of climate warming on LST trends remain ambiguous. Although the FVC has significantly declined in the western part of this region, there was a slight increase noted in the eastern part. Prior research has proposed that the decrease in FVC in the Sahel (the southern edge of the Sahara Desert) is due to reduced precipitation [7], a proposition that exhibits spatial inconsistency with our analysis. Furthermore, some studies have challenged the assertion that Sahel desertification can be solely attributed to decreased precipitation [38]. In some regions, FVC growth may be primarily attributed to large-scale ecological restoration and vegetation protection policies. For instance, measures implemented in China, such as the Grain for Green Program and ecological compensation initiatives, have facilitated grassland restoration and an increased FVC [39,40]. Similar efforts towards vegetation restoration and land conservation have been undertaken in numerous European countries to mitigate climate change and prevent soil degradation [41]. Additionally, in much of Europe, rising temperatures have allowed for the influx of moisture from the Mediterranean, creating favorable conditions for vegetation growth [42]. In some areas with appropriate climatic conditions, the ecosystem itself has a strong self-recovery capability, and FVC can be gradually restored, even without human activities. For instance, the western and southern parts of North America, characterized by relatively stable climate conditions and abundant water resources, enable the rapid recovery of grasslands from disasters [43,44].

In contrast, FVC showed a decreasing trend in southern Canada, the central United States (Great Plains region), and northern Australia (tropical grasslands and savanna regions), where the dominant causes were land use changes. The expansion of agriculture is the main driver of land use changes in southern Canada and central United States, leading to a reduction in natural grasslands and, consequently, a low FVC [45]. Similar land use transitions have occurred in northern Australia, where resource development activities may have further diminished grassland cover [46]. Additionally, northern Australia frequently experiences droughts driven by climate change, directly resulting in sparse grassland resources [47]. Extreme weather events such as wildfires and floods, which are becoming more frequent and severe in these regions, pose a challenge for the steady recovery of grassland ecosystems [48].

There were notable LST increases in northern Asia (Siberian tundra region), the boundary region between the Eurasian and African plates, eastern Africa, and central South America. In northern Asia, particularly in the area around the Arctic Ocean, the prominent temperature increase is closely related to global-warming-induced polar amplification (or Arctic amplification) [49,50]. This phenomenon has accelerated the rate of warming in high-latitude regions and exacerbated permafrost degradation, resulting in substantial carbon release and further LST increases [51,52]. In certain regions, such as eastern Africa and central South America, human-induced ecosystem changes have also contributed to the observed LST rise [53,54].

### 4.2. Analysis of Spatial Difference

**Figure 8:**
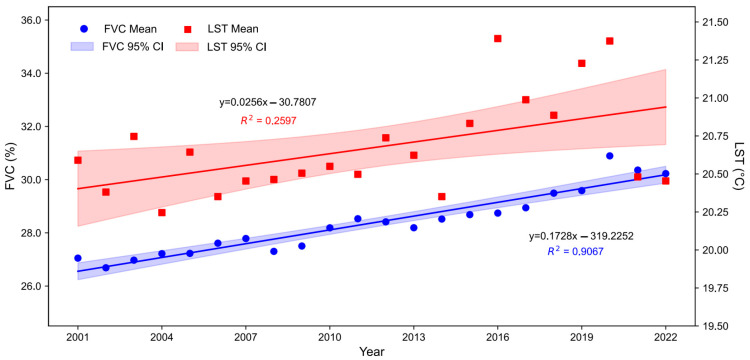
Mean FVC and LST of significantly changed grasslands from 2001 to 2022.

Figure 8 shows the trends of changes in the average FVC and LST over time for significantly changed grasslands from 2001 to 2022. Overall, the global average FVC and LST show an increasing trend, with FVC showing a better linear trend (R^2^ = 0.9067) than LST, which has a greater variability (R^2^ = 0.2597).

**Figure 9:**
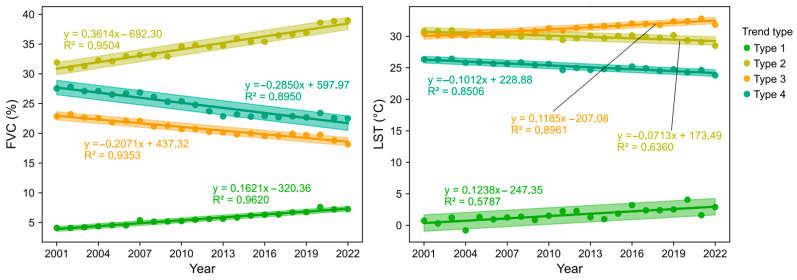
Time trends of FVC and LST under different increasing and decreasing trends. The shadow in the figure indicates a 95% confidence interval.

Figure 9 shows the time series of FVC and LST under different trend types, and intuitively shows the corresponding regression equations and R^2^ values of the four different trend types. The FVC under different trend types and the FVC of globally significant changing grasslands both have a good linear relationship, with R^2^ ≥ 0.8950. In contrast, the LST under different trend types shows a better linear fit with the LST of globally significant changing grasslands, with R^2^ ≥ 0.5787, even reaching 0.8961 under Type 3 conditions (decreasing FVC and increasing LST). This also indicates that, under certain conditions, more refined classification conditions can capture more accurate changes.

Among the four types of FVC and LST trends, Type 2 (FVC increase and LST decrease) accounts for the largest proportion, primarily distributed in the temperate grasslands of eastern North and South America, savannas of southern Africa, grasslands of central Asia, and tropical grasslands of northern Australia. Over the past two decades, these regions have experienced an average FVC increase of 8.80% ± 4.48% (*p* < 0.05) and an average LST decrease of 1.60 °C ± 1.26 °C (*p* < 0.05). Strong evidence supports the hypothesis that the LST reduction in these regions has been driven by the feedback regulation from the increased FVC (Spearman correlation = −0.63 ± 0.21). Research indicates that a surge in FVC typically results in a reduction in LST by amplifying latent heat flux (transpiration). This process is facilitated by the enhancement of surface roughness and conductivity, subsequently fostering turbulent flux dissipation. Nevertheless, this cooling impact might diminish in regions with limited water resources [55]. In low-latitude regions with sufficient water availability, an increase in FVC tends to result in a decrease in LST [56]. Hence, in Type 2 regions, especially those with tropical and subtropical humid climates, ecological restoration initiatives should prioritize maintaining an adequate water supply to guarantee successful vegetation rehabilitation.

The impact of FVC on LST in arid regions may differ from that in humid regions [8]. While a strong negative correlation is also observed (Spearman correlation = −0.67 ± 0.16), these areas are predominantly characterized by a decreasing FVC and increasing LST (Type 3). Such regions are mainly distributed in deserts and their surrounding areas, including the North American deserts, the Patagonian Desert, the Sahara Desert, the Turkestan Desert, the Taklamakan Desert, and the Australian deserts. Over the past two decades, these areas have experienced an average FVC decrease of 7.07% ± 6.04% (*p* < 0.05) and an average LST increase of 2.67 °C ± 1.59 °C (*p* < 0.05). Previous studies have indicated that an FVC decrease might cause a surface albedo decrease, and, thus, increase LST. At the same time, a decline in FVC might also cause a vegetation transpiration and soil evaporation decrease, leading to a surface moisture supply decrease, which might further affect LST [57,58]. Global-scale analyses by Duveiller et al. and Lian et al. emphasized the impact of vegetation changes on the surface energy balance, identifying the decline in vegetation cover in arid regions as a critical factor contributing to LST increases [59,60]. Moreover, studies have revealed that urban vegetation might reduce LST via shading and transpiration, thus playing a role in alleviating the urban heat island effect, which indicates that, in arid regions, an FVC decrease might also cause a transpiration decrease, and, thus, lead to a higher LST [61].

Type 1 (FVC increase and LST increase) exhibits distinct spatial characteristics, consistent with the findings of Myneni et al. in the late 20th century [62]. Vegetation growth in these regions is primarily concentrated in high-latitude (Latitude_abs = 55.27° ± 20.84°) and high-altitude (Elevation = 2198.14 m ± 1805.94 m) areas, including northern Asia, northern North America, and the Qinghai–Tibet Plateau. This spatial pattern has been confirmed by many studies, which believe that the greening of the Arctic caused by climate warming and the increasing concentration of CO_2_ can absorb more solar radiation, thus increasing the LST and forming a positive feedback mechanism that may intensify climate warming [63,64,65]. This phenomenon is reflected in the northward expansion of Arctic grasslands and boreal forests, supported by studies from Stow et al. [66] and Pearson et al. [67]. In the pan-third-pole region, permafrost degradation may increase surface water availability and greenhouse gas emissions, influencing vegetation growth. Simultaneously, LST increases extend the vegetation growing season [68,69]. However, due to the spatial heterogeneity of climate change in the Qinghai–Tibet Plateau, FVC and LST changes may exhibit varied patterns across regions [70,71], aligning with the findings of this study. Although the FVC generally increased across the third pole, certain areas, such as central Asia and Russia, showed a decline in FVC, highlighting that the trend of an increasing FVC is not uniform across all regions [72].

Type 4, characterized by a decreased FVC and a decreased LST, represents a mere 0.59% of global grasslands. This pattern is largely influenced by seasonal variations [73], ecosystem transitions (e.g., grasslands transitioning into shrublands), and human activities, with no discernible correlation between FVC and LST. While FVC showed an increasing trend in 80.69% of global grasslands, this trend remains unstable in high-latitude and high-altitude regions. Furthermore, degraded grasslands account for 19.31% of the global grassland area over the past two decades, highlighting the persistent issue of desertification. In these areas, the LST primarily exhibits an upward trajectory, potentially exacerbating the desertification process.

### 4.3. Analysis of Accuracy

When calculating the grassland range, the maximum area where grassland has appeared more than five times or has existed continuously for more than 3 years at the same coordinate point from 2001 to 2022 was used as the grassland range, ignoring the dynamic process of the grassland. This calculation often makes the grassland range not very accurate.

The analysis of FVC and LST trends primarily relies on remote sensing data, which may be limited by data quality and resolution. The annual mean NDVI and LST used in this study were calculated by averaging monthly data for each year to assess global trends in FVC and LST changes. Although cloud masking algorithms were employed, the influence of dense cloud cover remained unavoidable. Additionally, factors such as snow cover, atmospheric aerosols, and extreme weather events often cause monthly NDVI and LST values to deviate from their standard values [74], potentially affecting the observed trends. Other disturbances, such as forest fires, floods, droughts, volcanic eruptions, and human activities, also impact the development of NDVI and LST trends [75]. However, this study did not account for these factors. Moreover, the analysis exclusively relied on spatial data of annual mean FVC and LST, which may overlook the impact of seasonal variations [76].

In arid and semi-arid regions, where the vegetation cover density is low, the surface reflectance is relatively high, resulting in NDVI values that are lower than their actual values. Consequently, the FVC trends in these areas may be influenced by unaccounted soil reflectance [29]. Additionally, factors such as soil moisture, atmospheric conditions, and biodiversity, which could affect FVC and LST, were not considered in this study. Future research should integrate a wider range of data and methodologies to achieve a more comprehensive understanding of FVC and LST trends and their driving mechanisms.

The algorithms used in this study, including Mann–Kendall trend analysis and Spearman correlation, were applied uniformly across all regions. However, their applicability in high-altitude areas may be influenced by the unique climatic and ecological conditions. For example, the accuracy of LST retrieval algorithms can be affected by snow and ice cover, while FVC measurements may be less reliable in areas with sparse vegetation. These factors could introduce uncertainties in the results for regions like the Tibetan Plateau or the Andes.

## 5. Summary and Conclusions

This study leveraged long-term remote sensing data (2001–2022) to scrutinize the trends and interrelationships between FVC and LST in global grassland ecosystems. The Mann–Kendall trend test and Spearman correlation analysis were employed as analytical tools. The findings reveal a marked spatial heterogeneity, with the trends of FVC and LST exhibiting regional variations.

On a global scale, both the FVC and LST trends exhibited regional differentiation. There was an increase in the FVC across eastern Eurasia, eastern South America, western and southern North America, and central China. Conversely, a decrease was observed in southern Canada, the central United States, and northern Australia. In terms of LST, warming trends were prevalent in North Asia, the Qinghai–Tibet Plateau, central and southern South America, and Africa. However, cooling trends were evident in the Andes, southern Sahara, and eastern Australia. Overall, both the global FVC and LST were on the rise, with FVC displaying a more pronounced linear trend (R^2^ = 0.9067) compared to LST (R^2^ = 0.2597).

Based on the observed trends, global grasslands were classified into the following four distinct categories: Type 1 (increased FVC and increased LST), Type 2 (increased FVC and decreased LST), Type 3 (decreased FVC and increased LST), and Type 4 (decreased FVC and decreased LST). Time series analysis indicated that FVC exhibited a robust linear trend (R^2^ ≥ 0.8950), while the linear trend of LST showed an improved consistency within these classified regions (R^2^ ≥ 0.5785). This suggests that more refined classifications offer superior insights into the observed changes.

Among the four types, Type 2 accounted for the largest proportion (46.98%) and was mainly distributed across the temperate grasslands of eastern North and South America, savannas of southern Africa, central Asia, and northern Australia, where FVC increased and latent heat flux, transpiration, and surface roughness were enhanced, thus reducing LST. Type 3 accounted for 18.72% of the total area and was mainly distributed in arid areas such as North American deserts, the Sahara, and Australian deserts, where a low FVC weakened transpiration and increased LST. Type 1 accounted for 33.72% of the total area and was mainly distributed in high-latitude and high-altitude regions (e.g., North Asia, northern North America, and the Qinghai–Tibet Plateau), where increased vegetation growth intensified LST warming. Type 4 had the smallest area (0.59%) and was mainly affected by seasonal variation; this type showed no significant correlation between FVC and LST.

Future research should concentrate on the enduring effects of climate change on grasslands and emerging ecological challenges. Important areas to explore include feedback mechanisms between vegetation cover, surface temperature, and climate variability—particularly the impact of permafrost degradation in high-latitude regions on ecosystem stability. Incorporating seasonal variations and extreme weather events is vital for understanding both short-term fluctuations and long-term trends. In regions where water resources are limited, integrating precipitation and soil moisture data will enhance our understanding of the factors influencing vegetation dynamics and surface temperature changes. This knowledge is critical for developing water conservation strategies and drought-resistant crop plans, thus mitigating the effects of water scarcity on grasslands. For high-altitude regions, tactics to mitigate surface warming from permafrost degradation should prioritize promoting vegetation cover to boost insulation, reduce soil erosion, and manage greenhouse gas emissions from thawing permafrost.

## Figures and Tables

**Table 1:** Distribution of data under different increasing and decreasing trends of FVC and LST.

Trend Type	FVC_TS Slope		LST_TS Slope		Spearman Correlation		Elevation (m)		Latitude_abs (°)	
	Mean	Std.	Mean	Std.	Mean	Std.	Mean	Std.	Mean	Std.
1	0.002322	0.001219	0.121369	0.080988	0.649541	0.390623	2198.14	1805.94	55.27	20.84
2	0.004002	0.002200	−0.072790	0.057298	−0.625784	0.211534	859.98	806.06	24.45	13.63
3	−0.003213	0.002747	0.121344	0.072349	−0.665806	0.159668	586.37	644.62	21.00	15.07
4	−0.003450	0.004297	−0.110851	0.144191	0.079621	0.556339	835.98	1056.23	26.74	18.83

**Table 2:** Overview of remote sensing dataset information.

Data Type	GEE Product Number	Minimum Resolution (m)	Obtain Time Range
NDVI	MODIS/006/MOD13Q1	250	2001–2022
Landcover	MODIS/061/MCD12Q1	500	2001–2022
LST	MODIS/061/MOD11A1	1000	2001–2022
DEM	COPERNICUS/DEM/GLO30	30	-

## Data Availability

Data are contained within the article.
